# Inflammatory Gene Expression Associates with Hepatitis B Virus cccDNA- but Not Integrant-Derived Transcripts in HBeAg Negative Disease

**DOI:** 10.3390/v14051070

**Published:** 2022-05-17

**Authors:** Andrea Magri, James M. Harris, Valentina D’Arienzo, Rosalba Minisini, Frank Jühling, Peter A. C. Wing, Rachele Rapetti, Monica Leutner, Barbara Testoni, Thomas F. Baumert, Fabien Zoulim, Peter Balfe, Mario Pirisi, Jane A. McKeating

**Affiliations:** 1 Nuffield Department of Medicine, University of Oxford, Oxford OX3 7FZ, UK; andrea.magri@ndm.ox.ac.uk (A.M.); james.harris@ndm.ox.ac.uk (J.M.H.); valentinada83@gmail.com (V.D.); peter.wing@ndm.ox.ac.uk (P.A.C.W.); peter.balfe@ndm.ox.ac.uk (P.B.); 2 Department of Translational Medicine, Università del Piemonte Orientale, 28100 Novara, Italy; rosalba.minisini@med.uniupo.it (R.M.); rachele.rapetti@uniupo.it (R.R.); mario.pirisi@med.uniupo.it (M.P.); 3 Institut de Recherche sur les Maladies Virales et Hépatiques, University of Strasbourg and Inserm, UMR_S1110, F-67000 Strasbourg, France; juehling@unistra.fr (F.J.); thomas.baumert@unistra.fr (T.F.B.); 4 Chinese Academy of Medical Sciences Oxford Institute, University of Oxford, Oxford OX1 4BH, UK; 5 Department of Diagnostic Services and Supportive Therapies, ASL Verbano-Cusio-Ossola, 28887 Omegna, Italy; leutner@aslvco.it; 6 Cancer Research Center of Lyon, UMR INSERM 1052, 69008 Lyon, France; barbara.testoni@inserm.fr (B.T.); fabien.zoulim@inserm.fr (F.Z.); 7 Pôle Hépato-Digestif, Institut Hopitalo-Universitaire, Hôpitaux Universitaires de Strasbourg, 67000 Strasbourg, France; 8 Institut Universitaire de France, 75005 Paris, France

**Keywords:** hepatitis B virus, cccDNA, transcription, inflammation

## Abstract

Chronic hepatitis B virus (HBV) infection is a global health problem that presents as a spectrum of liver disease, reflecting an interplay between the virus and the host immune system. HBV genomes exist as episomal covalently closed circular DNA (cccDNA) or chromosomal integrants. The relative contribution of these genomes to the viral transcriptome in chronic hepatitis B (CHB) is not well-understood. We developed a qPCR method to estimate the abundance of HBV cccDNA- and integrant-derived viral transcripts and applied this to a cohort of patients diagnosed with CHB in the HBe antigen negative phase of disease. We noted a variable pattern of HBV transcripts from both DNA templates, with preS1/S2 mRNAs predominating and a significant association between increasing age and the expression of integrant-derived mRNAs, but not with inflammatory status. In contrast, cccDNA-derived transcripts were associated with markers of liver inflammation. Analysis of the inflammatory hepatic transcriptome identified 24 genes significantly associated with cccDNA transcriptional activity. Our study uncovers an immune gene signature that associates with HBV cccDNA transcription and increases our understanding of viral persistence.

## 1. Introduction

Hepatitis B virus (HBV) is a leading cause of viral hepatitis with more than 240 million infections resulting in an estimated 780,000 deaths a year from liver disease and hepatocellular carcinoma (WHO, Global Hepatitis Report 2017). Immunological control of HBV is complex, involving both the innate and adaptive immune systems, yet chronic hepatitis B (CHB) is often associated with an exhausted antiviral immune response [1,2]. HBV is the prototype member of the *hepadnaviridae* viral family, which replicate via episomal copies of a covalently closed circular DNA (cccDNA) genome. This complexes with histone and non-histone proteins to resemble cellular chromatin. The cccDNA transcription is regulated by host transcription factors, co-activators, co-repressors, and chromatin modifying enzymes [3]. Interferons have been shown to inhibit cccDNA transcriptional activity by altering histone hypoacetylation and by recruiting transcriptional corepressors [4,5]. HBV is recognised to evade innate immune recognition, resulting in a muted antiviral response in CHB [6,7], highlighting our limited understanding of the endogenous immune pathways that regulate cccDNA transcription. Although the level of cccDNA in the infected liver is low, with as few as 1–3 copies per infected hepatocyte, its long half-life supports persistent infection [8,9]. The size and transcriptional activity of the cccDNA pool is thought to be the major determinant of HBV replication [10].

HBV transcribes six major viral RNAs of decreasing length with a common 3′ polyadenylation signal together with a number of spliced RNAs of unknown function [11]. The major RNAs include: precore (pC), which encodes e antigen (HBeAg); pregenomic (pg RNA), which is translated to yield core protein or antigen (HBcAg) and polymerase; and preS1, which encodes large surface envelope (L-HBsAg) and S RNA, and encodes medium (M-HBsAg; Pres1 and S), and small surface envelope (S-HBsAg, S) glycoproteins (HBsAg) and the X transcript codes for the multi-functional x protein (HBx). Encapsidated pg RNA is reverse-transcribed by the viral polymerase to generate new DNA genomes that may be reimported to the nucleus to maintain the cccDNA pool or secreted as infectious particles. Aberrant reverse transcription of pg RNA can generate a double-stranded linear DNA (dslDNA) that may integrate into the host genome [12]. This integrated DNA (iDNA) is permuted such that the basal core promoter (BCP) is downstream of the major open reading frames so that neither pC nor pg RNAs are transcribed; however, the iDNA can act as a template for HBsAg and HBx expression. Viral integrants have been reported to drive HBsAg expression [13,14]; however, the relative contribution of cccDNA or iDNA templates to the viral transcriptome in chronic HBeAg negative patients is not well-understood.

CHB presents as a spectrum of disease and can be classified into four clinical phases defined by peripheral viral, biochemical, and histological features [15]. However, this simple classification does not reflect the status of infected cells or immune responses in the liver. Early in infection, cccDNA is transcriptionally active and pC/pg RNAs drive viremia and HBeAg expression. However, in later stages of chronic infection, after HBeAg seroconversion and the loss of antigen (HBeAg-negative), peripheral HBV DNA levels are reduced that may reflect either the loss or reduced transcriptional activity of cccDNA [10,16]. The majority of subjects diagnosed with CHB are HBeAg-negative and often suppress virus replication for many years. HBV can reactivate spontaneously or following immunosuppressive or anti-inflammatory therapies [17]; however, the immune pathways controlling virus replication are not well-defined and require further study.

Current nucleos(t)ide analogue (NA) therapies suppress cytoplasmic HBV replication but do not target cccDNA and hence are not curative [18]. In this study we report on the development of a PCR-based assay to discriminate between cccDNA and integrant derived RNAs and its application to study the interplay between HBV transcription and hepatic inflammatory responses.

## 2. Materials and Methods

### 2.1. Liver Biopsies, RNA Extraction and HBV Antigen Immunohistochemistry

Liver biopsies from chronic hepatitis B (*n* = 26) and non-infected subjects (*n* = 8) were collected with informed consent to have a small part of their biopsy specimen, exceeding that needed for complete pathology examination, used for research purposes. The local ethical committee approved the use of this archival material (CE90/19) together with anonymised clinical and demographic data for the purposes of this study. Samples of each biopsy were stored in RNAlater (Thermo Fisher, Waltham, MA, USA) at −80 °C or fixed in 10% buffered formalin. Paraffin-embedded sections (5 μm) were stained with hematoxylin and eosin, Masson’s trichrome, and Gomori’s silver impregnation for reticulin fibres. Histological grading and staging scores were determined according to the Ishak scoring system [19]. Immunohistochemistry was performed on sections of fixed liver biopsies with a commercial streptavidin–biotin technique according to the manufacturer’s instructions on a BENCHMARK XT staining system (Ventana Medical Systems, Tucson, AZ, USA) using primary mouse monoclonal antibodies against HBcAg (clone LF161, Novocastra TM IHC Antibodies, Leica Biosystems, Wetzlar, Germany) or HBsAg (clone 3E7, Santa Cruz Biotechnology, Dallas, TX, USA).

### 2.2. Peripheral HBsAg Quantification

HBsAg was quantified from plasma using the fully integrated LIAISON-XL Murex HBsAg Quant chemiluminescence system (DiaSorin, Saluggia, Italy).

### 2.3. RNA and DNA Extraction

Liver biopsy fragments were mechanically disaggregated in Trizol and RNA extracted from the aqueous phase by chloroform phase separation and isopropanol precipitation. DNA was extracted by ethanol precipitation from the organic phase, following the manufacturer’s instruction (Thermo Fisher). RNA concentration and integrity were assessed by RNA ScreenTape analysis on a 2200 TapeStation (Agilent, Santa Clara, CA, USA).

### 2.4. Quantification of HBV Transcripts

For each sample, 1 µg of RNA was digested with 2 units of TurboDNAse (Thermo Fisher) for 30 min at 37 °C, treated with a DNAse inactivation reagent, centrifuged for 1.5 min at 10,000× *g* and RNA collected from the aqueous phase. 500 ng of RNA was reverse transcribed using random hexamers with a SuperScript III RT Kit (Life Technologies, Carlsbad, CA, USA), following the manufacturer’s protocol. PCR reactions with the previously reported T1-T4 (in this study T4 is referred to as 3′T) [20] and RR primers (RR forward: TTTCACCTCTGCCTAATCATCTCT; RR reverse: CTTTATAAGGGTCAATGTCCATGC) were performed using a SYBR green protocol (qPCRBIO SyGreen, PCR Biosystems, London, UK) in a Lightcycler 96™ instrument (Roche, Basel, Switzerland). The amplification conditions were: 95 °C for 2 min, followed by 45 cycles of amplification (95 °C for 5 s; 60 °C for 30 s). DNase-treated RNA samples that had not been reverse-transcribed were amplified to verify the absence of residual DNA. Viral RNA copies were quantified using a standard curve of 10-fold serially diluted plasmid encoding a single copy of the HBV GtD genome (HBV 1.0), which showed a linear amplification below a Ct value of 31. Primers are designed to co-amplify multiple transcripts: T1 for pC/pgRNA; T2 for pC/pgRNA and preS1; T3 for pC/pgRNA, preS1, and preS2; and T4 (3′T) to amplify all the major viral transcripts. To enumerate individual transcript copies, subtraction of the inferred copy number for selected primers was used to ascribe the relative copy number for each contributing RNA, as previously reported [20]. Determining the relative expression of the major viral RNAs using raw Ct values or inferred viral RNA copy numbers showed comparable results (Supplementary Figure S1A). Copy number enumeration from random hexamer-based RT or from an oligo-dT/random hexamers-based RT showed comparable results (Supplementary Figure S1B). cccDNA-derived transcripts were measured by qPCR using the RR primer pair that amplifies a region occurring in both the 5′ and the 3′ termini of the pC and pg ~3.5 Kb RNA transcripts. These primers will therefore overestimate pC/pg RNAs relative to other transcripts. Ct values were adjusted for RNA quantity using two housekeeper genes (Rplp0 and B-Act) and relative amounts of total and cccDNA-derived RNA estimated using the formula: ∆Ct = Ct_RR_ − Ct_3′T_.

### 2.5. Quantification of HBV DNA

Total hepatic HBV DNA was amplified using RR primers and copy number inferred from a standard curve obtained from 10-fold serial dilutions of a plasmid containing a single copy of HBV DNA. Copy numbers were adjusted for input control prion protein (PrP). The cccDNA was quantified from biopsy DNA with digital droplet PCR as previously reported [16,21,22].

### 2.6. Nanostring Inflammatory Gene Analysis

50 ng of each biopsy RNA were incubated with a nanostring inflammation panel of 249 inflammation-related genes at 65 °C for 16 h. Samples were diluted in DEPC-water, loaded into cartridges, and assessed using an nCounter fluorescence detector. Gene expression values were normalised using the geometric mean of 6 calibrator housekeeping genes (CLTC, GAPDH, GUSB, HPRT1, PGK1, TUBB) using nSolver software and data deposited at Gene Expression Omnibus under the accession GSE169110. Genes that were expressed in all CHB samples (*n* = 87) were compared with pC/pg, preS1/S2 RNA and RR values by Spearman rank correlation and significant results (*p* < 0.05) corrected for 5% FDR using the two-stage step-up method of Benjamini, Krieger, and Yekutieli.

### 2.7. Pathway Analysis

Pathway analysis was carried out using PantherDB where overrepresentation of genes was assessed for gene ontology (GO) biological processes, using Fisher’s Exact test with an FDR cut-off of 0.05. Pathways were refined using REVIGO with a 0.5 cut-off value for redundancy, and processes in which at least 50% of genes were represented. Fold enrichment was log_2_ transformed and values >100 plotted as log_2_(100).

### 2.8. Single Cell RNA-Seq Analysis

Single cell data was processed as described previously [23]. We used the “plotexpmap” function of the RaceID3 package in R to generate the figure showing log2-transformed expression values in the human liver single cell atlas [23].

### 2.9. Statistics

For all analyses the individual replicate numbers are provided in the figure legends. Statistical assessments were performed using ANOVA (for more than two group comparisons), Mann–Whitney tests (two group comparisons; unpaired data) or Wilcoxon tests (two group comparison; paired data) using Prism 9.3.1 (GraphPad, San Diego, CA, USA). Principal component analysis and unsupervised hierarchical clustering were performed on gene expression levels standardised by Z-score transformation using Prism 9.3 (GraphPad) and R 4.1 respectively. In the figures * denotes *p* < 0.05, ** *p* < 0.01, *** *p* < 0.001, and **** *p* < 0.0001.

## 3. Results

### 3.1. Increased HBV Transcription in Active Hepatitis

**Figure 1:**
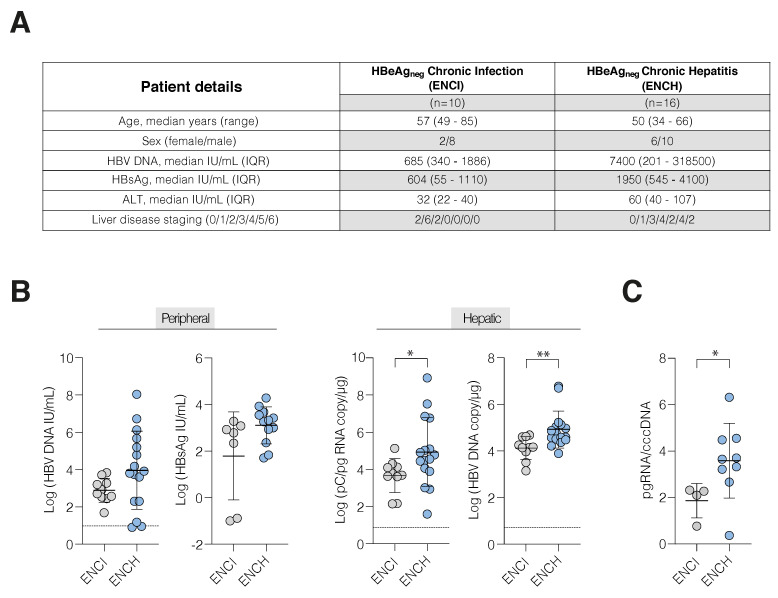
HBV replication in chronic disease. (**A**) Clinical parameters of HBeAg-negative CHB in patients diagnosed with chronic infection (ENCI, *n* = 10) or chronic hepatitis (ENCH, *n* = 16) according to EASL guidelines. (**B**) Peripheral HBV DNA, HBsAg, and hepatic pC/pg RNA and HBV DNA levels in ENCI (grey symbols) and ENCH (blue symbols). HBsAg only available for 19 samples. (**C**) The cccDNA transcriptional activity measured as the ratio of pC/pg RNA to cccDNA. Data are presented as the mean copy number derived from two technical replicates and compared using a Mann–Whitney U-test (* *p* < 0.05, ** *p* < 0.01). The dashed line denotes the cut-off of the assays.

Peripheral and hepatic markers of HBV replication were measured in a cohort of 26 treatment-naïve subjects with HBeAg-negative chronic infection (ENCI, *n* = 10) or chronic hepatitis (ENCH, *n* = 16) in accordance with current EASL guidelines [15] (Figure 1A and Supplementary Table S1). We noted a >7-log range in peripheral HBV DNA levels, from undetectable (<10 IU/mL) to 1.1 × 10^8^ IU/mL, with higher viremia in ENCH; however, comparable amounts of peripheral HBsAg were seen in both disease groups (Figure 1B), in line with previous reports [10,16]. As expected, ENCH patients exhibited higher alanine aminotransferase (ALT) levels and more advanced liver disease (Supplementary Table S1). To assess whether the wide-range in peripheral HBV DNA reflects differences in viral transcription and/or replication in the liver we quantified viral RNA and DNA by qRT-PCR. Since the BCP drives transcription of pC and pg RNA from two start sites that are only 70 base pairs apart, and our qPCR cannot discriminate between them, we defined this amplicon as pC/pg. Prakash et al. reported that pg RNA was 100-fold more abundant than pC RNA in HBeAg-positive patients, however, the difference was modest in HBeAg negative patients [24]. Higher levels of pC/pg RNA and HBV DNA were seen in biopsies from patients with hepatitis (ENCH) (Figure 1B). Quantifying cccDNA using an established digital droplet PCR [16,21,22] identified thirteen samples that were below the limit of detection; however, the remaining samples amplified. We observed higher pC/pg RNA relative to cccDNA in ENCH (Figure 1C) and an increase, although not significant, in cccDNA copies (Supplementary Figure S2), suggesting that the higher viremia in subjects with hepatitis reflects increased cccDNA transcription.

**Figure 2:**
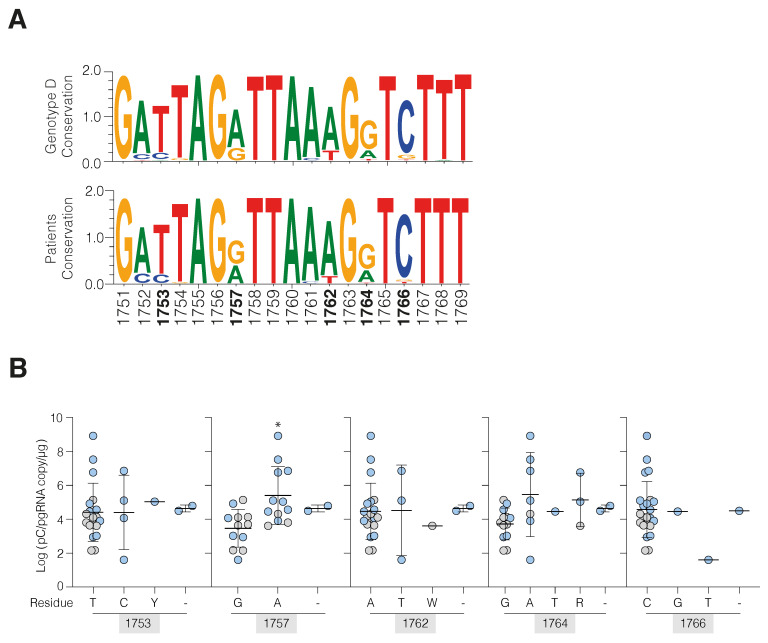
Basal core promoter polymorphisms and pC/pg RNA levels. (**A**) Consensus plot of the BCP region (1751–1769) of 1202 HBV genotype D sequences (HBV data base) and 26 CHB patient-derived sequences showing genetic variability. (**B**) Patients were grouped by the consensus nucleotide at previously reported residues of interest (1753, 1757, 1762, 1764, and 1766). Symbols show the mean pC/pg RNA copy number derived from two technical replicates where symbols represent ENCI (grey symbols) and ENCH (blue symbols). Differences were assessed using Kruskal–Wallis ANOVA with Dunn’s multiple comparison correction (* *p* < 0.05).

Since genetic polymorphisms in the BCP may explain variable pC/pg RNA levels [25] we sequenced a 600bp DNA amplicon spanning the promoter boundaries. Bulk sequencing typically detects polymorphic residues when present at >10%. The consensus for our patients matched the HBV database for genotype D, although 2/26 patients showed a partial or complete deletion of the BCP region (Figure 2A and Supplementary Figure S3). Nucleotides at positions 1753, 1757, 1762, 1764, and 1766 were reported to associate with transcriptional activity [26,27] and we assessed the relationship between these polymorphisms and pC/pg RNA levels. With the exception of G1757A, we found limited evidence of an association between these polymorphic residues and pC/pg RNA levels (Figure 2B). We noted higher pC/pg RNA in biopsy samples with 1757A (median of 4.68 × 10^3^ for G and 7.2 × 10^4^ for A, *p* < 0.01 Figure 2B). The G1757A polymorphism was reported to associate with the double substitution 1764T/1766G [28] and we identified this double mutant in one patient. However, none of the double substitutions associated with pC/pgRNA levels. These data suggest that polymorphisms within the BCP do not explain the variation in pC/pg RNA levels, supporting a model of increased cccDNA activity during periods of hepatitis.

### 3.2. HBV cccDNA and Integrant-Derived RNAs and Antigen Expression

**Figure 3:**
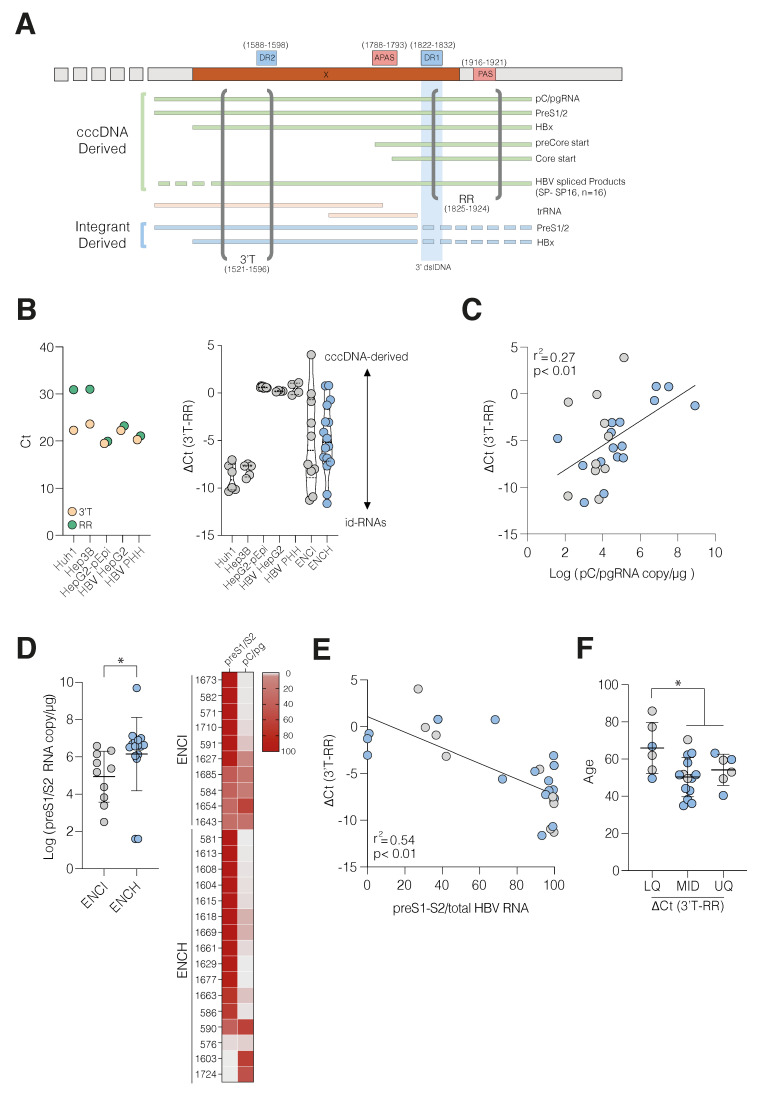
Quantification of HBV cccDNA and integrant-derived transcripts. (**A**) Graphic depicting HBV cccDNA- and integrant-derived transcripts; location of HBx ORF (X); direct repeats 1 and 2 (DR1 and DR2); the primary and alternative (cryptic) polyadenylation signals (PAS and APAS); 3′ end of double stranded linear genome (3′ dslDNA) and the regions amplified by 3′T and RR primers with coordinates shown. (**B**) PCR Ct values of 3′T or RR primers on amplification of cDNA isolated from HBV integrant lines (Hep3B, Huh-1, *n* = 6); HepG2 with episomal HBV DNA (HepG2-pEpi, *n* = 4); HBV-infected HepG2-NTCP cells or PHHs (3 days post-infection, *n* = 4) (**left**). ΔCt (3′T-RR) of cell culture systems and CHB liver biopsies from ENCI (grey symbols) or ENCH (denoted in blue symbols). Each symbol represents the mean value from 2 technical PCR replicates, where the median and quartiles are shown (**right**). (**C**) Association between pC/pg RNA and ΔCt (3′T-RR) value was assessed using Spearman correlation coefficient. (**D**) preS1/S2 RNA copies in ENCI and ENCH (**left**) and relative pC/pg and preS1/S2 RNA levels in CHB expressed as a percentage of the total RNA (**right**), where the scale bar depicts the abundance of each transcript. (**E**) Association between preS1/S2 RNA and ΔCt (3′T-RR) value was assessed using Spearman’s correlation coefficient. (**F**) Association between patient age at the time of biopsy and ΔCt (3′T-RR) value. Patients were classified by ΔCt (3′T-RR) value based on the 25% (LQ); 25–75% (MID) and 75% (UQ) quartiles and significance assessed using a Mann–Whitney U-test comparing the LQ with MID and UQ groups (* *p* < 0.05).

To study the transcriptional activity of episomal cccDNA and iDNA we used a qPCR method to quantify transcripts from viral genomes. Primers targeting the 3′ end of all transcripts (3′T) will amplify both cccDNA- and iDNA-derived RNAs, whereas those targeting the repeat region (RR), which is absent in the majority of id-RNAs, will only amplify cccDNA-derived RNAs [14,29] (Figure 3A). The 3′T primers will amplify pC/pg splice variants [11] and two of the three known HBx truncated transcripts [30]. Both 3′T and RR primer pairs target regions conserved among diverse genotypes and showed a linear amplification of HBV genotype D over a 5-log range (Supplementary Figure S4A,B). Biopsy-derived cDNA showed a similar amplification profile (Supplementary Figure S4C). As the RR target is present in both the 5′ and 3′ ends of the pC/pg RNA these primers will give a higher signal from cccDNA-derived targets than the 3′T primers. To validate this approach, we selected two well-characterised hepatoma lines, Huh1 and Hep3B, that harbour transcriptionally active integrated genomes [31] and HepG2 cells bearing an episomal-replicating HBV (HepG2-pEpi) [32]. RNA from both integrant lines amplified with 3′T (Ct between 22–23) but not with the RR primers (Ct values around 30–33, with ∆Ct values of 10) (Figure 3B, left), demonstrating primer specificity. Whereas both primer pairs amplified viral RNAs from HepG2-pEpi, de novo HBV infected HepG2-NTCP cells and primary human hepatocytes (PHH) with similar Ct values (Figure 3B, left). The difference in PCR amplification threshold (∆Ct) between the 3′T and RR primers estimates the relative abundance of cccDNA-derived RNAs (Figure 3B, right). The CHB biopsies showed a wide range of ∆Ct values, consistent with transcription from both cccDNA and iDNA (Figure 3B, right). Since pC/pg RNA can only be transcribed from cccDNA, it was reassuring to observe an association with ∆Ct (3′T-RR) value (r^2^ = 0.27 and *p* < 0.01, Figure 3C) and RR-inferred viral transcripts (r^2^ = 0.62, *p* < 0.01, Supplementary Figure S4D).

Recent studies show that iDNA is the major source of HBsAg [10,14] leading us to estimate preS1/S2 transcript levels [20]. Elevated preS1/S2 RNA levels were seen in ENCH (Figure 3D, left), with these transcripts comprising >95% of viral RNA in 18/26 of biopsies (Figure 3D, right). The relative abundance of preS1/S2 RNAs showed an inverse relationship with the ∆Ct (3′T-RR) value, demonstrating that patients with fewer cccDNA-derived transcripts have a higher percentage of preS1/S2 RNAs (Figure 3E). Eight biopsies showed similar levels of pC/pg and preS1/S2 RNAs, consistent with a lower ∆Ct (3′T-RR) value (median −0.24) compared to the other samples (median −2.12; *p* < 0.01) and an estimated 4-fold increase in cccDNA activity. HBV integration events are common in CHB and are linked to HCC [12,33,34]; however, the relationship between patient age and iDNA transcriptional activity is not well-understood. Stratifying patients by ∆Ct (3′T-RR) values showed an association with age for subjects in the lowest quartile (*p* < 0.01) (Figure 3F). Collectively, these data suggest that iDNA is the predominant source of viral transcripts in older patients.

**Figure 4:**
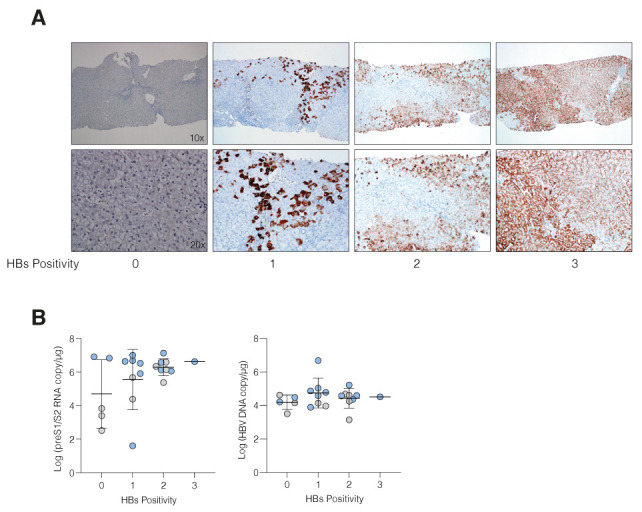
HBsAg liver staining and preS1/S1 RNA levels. (**A**) HBsAg immune staining in liver biopsies. Samples were scored based on the frequency of HBsAg-expressing cells, where 0 is negative, 1 is less than 33%, 2 between 33–66%, and 3 higher than 66%. Two magnifications are shown: 10× (**top**) and 20× (**bottom**). (**B**) Association between HBsAg score and preS1/S2 RNA copies (**left**) or total hepatic DNA (**right**).

To determine whether a high frequency of preS1/S2 RNAs in the liver associates with viral antigen expression we stained sequential biopsy sections for HBsAg. Immunostaining data were available for 22 subjects, with 17 samples staining for HBsAg (ENCI *n* = 6/9, ENCH *n* = 11/13) and a subset (*n* = 4) positive for HBcAg (ENCI *n*= 0/9, ENCH *n* = 4/13). We noted a high frequency of HBsAg-expressing cells, which appeared in discrete clusters, with many biopsies expressing HBsAg in >33% of hepatocytes (Figure 4A). In stark contrast, HBcAg-expressing hepatocytes were infrequent and generally observed as isolated single cells (Supplementary Figure S5). Stratifying biopsies according to their frequency of HBsAg^pos^ hepatocytes (0 = negative; 1 <33%; 2 = 33–66% and 3 >66% HBsAg^pos^ hepatocytes) showed a trend towards preS1/S2 RNA levels associating with higher HBsAg scores independent of disease stage (Figure 4B). There was no link between the hepatic HBsAg score and peripheral HBV DNA levels, consistent with the limited evidence of hepatocytes expressing both HBsAg and HBcAg and the chimeric nature of the liver with rare hepatocytes supporting cccDNA replication (Figure 4B). These data show that preS1/S2 RNAs and the frequency of HBsAg expressing hepatocytes are independent of disease staging.

### 3.3. Active HBV Replication Associates with Inflammatory Gene Expression

**Figure 5:**
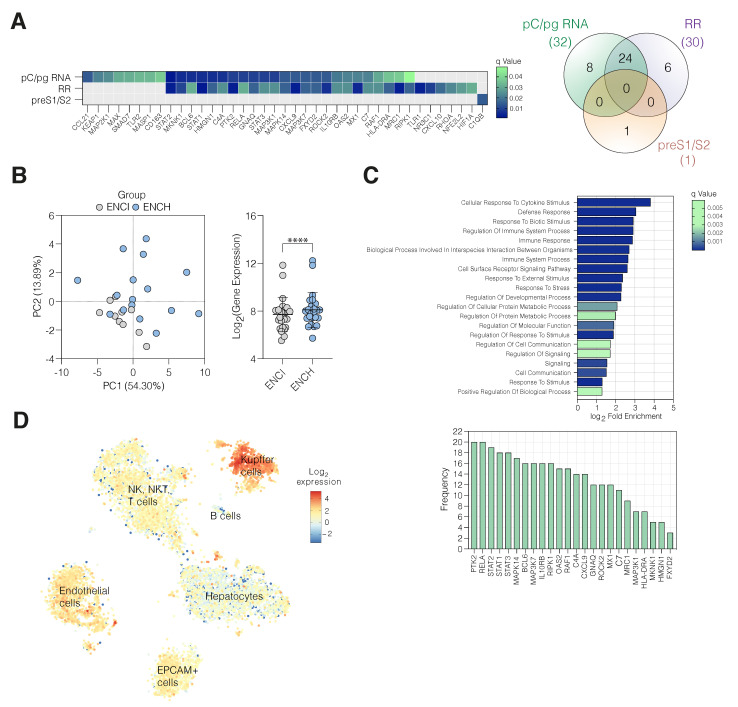
HBV transcripts and inflammation. (**A**) Heatmap and Venn diagram depicting the q-value and inflammatory gene transcripts that positively associate with pC/pg, RR, or preS1/S2 RNAs from CHB biopsies and their overlap. Probabilities were estimated using Spearman’s rank correlation coefficient with *p*-values corrected for a 5% false discovery rate (FDR) using the two stage Benjamini–Krieger–Yekutieli method. (**B**) Principal component analysis of the 24 genes associated with pC/pg RNA and RR identified in (**A**) stratifies patients according to their EASL disease staging (ENCI- grey and ENCH- blue). Average expression of the 24 gene signature in biopsies from patients diagnosed as ENCI or ENCH (Wilcoxon signed-rank test, **** *p* < 0.0001). (**C**) Pathway analysis of enriched GO biological processes were determined using PantherDB and redundancy accounted for using REVIGO. 21 pathways were identified where at least half of our gene set was represented. (**D**) Cell-type-specific expression of the 24 inflammatory genes in the human liver single cell atlas [23], where data is presented as the mean of the log2-transformed values.

Our data show clear evidence for higher cccDNA transcription in ENCH; however, the underlying immune pathways are not well-understood. To explore this, we quantified 249 host gene transcripts mapping to inflammatory pathways in liver biopsy samples by nCounter. As an internal control we designed custom Nanostring probes targeting the HBV 3′T amplicon and observed a significant correlation with qPCR-measured HBV RNA copies (r^2^ = 0.8, *p* < 0.0001). Eighty seven inflammatory gene transcripts were detected in all CHB samples, and 32 positively correlated with pC/pg RNA and a subgroup of 24 genes with viral RNAs amplified by the RR primers (Spearman’s correlation coefficient, q-value for FDR < 0.05) (Figure 5A). In contrast, only a single inflammatory transcript (C1QB) associated with preS1/S2 RNA and showed no overlap with the 24 genes linked with cccDNA-derived RNAs (Figure 5A). None of the inflammatory genes showed a negative association with any viral parameters, consistent with limited immune control of viral transcription during chronic infection. To focus on the immune genes that associate with cccDNA transcription we selected the 24 genes that associate with both pC/pg and RR amplification (Figure 5A). The 24-gene set was expressed at higher levels in ENCH versus ENCI and principal component analysis and unsupervised hierarchical clustering partitioned the cohort according to disease staging (Figure 5B, Supplementary Figure S6). Pathway analysis of the genes highlighted their involvement in the defense response, stress response, as well as immune system processes and the cellular response to cytokine stimulus (Figure 5C) [35]. All of the 24 gene transcripts detected in CHB were expressed in biopsies from control nonviral infected subjects, allowing us to assess the cell types within a healthy liver that express these genes (Supplementary Figure S7). Analysing a liver single cell atlas database [23] identified the highest expression in Kupffer cells (KC) and sinusoidal endothelial cells (Figure 5D). Collectively these studies show evidence of an inflammatory signature associating with cccDNA transcription.

## 4. Conclusions

CHB represents a spectrum of disease that reflects a dynamic interaction between the virus and the hepatic immune system. In the majority of HBeAg-negative subjects we studied, >95% of viral transcripts were preS1/S2 RNAs that most likely derive from viral integrants. These data are in agreement with Prakash et al., who reported that 90% of S RNA in HBeAg-negative patients derived from iDNA [24]. This pattern of viral RNA is reflected by the detection of HBsAg-expressing hepatocytes in liver biopsies and is consistent with published studies [12,14,34]. In contrast, HBcAg^pos^ hepatocytes were rare and only found in subjects with active hepatitis. These HBcAg^pos^ hepatocytes were randomly distributed across the biopsy, consistent with a cell-intrinsic property that supports cccDNA transcription. Our PCR approach to estimate the source of HBV transcripts identified patients with transcriptionally active cccDNA and highlights the value of using spatial in situ sequencing approaches to identify the transcriptomes of permissive hepatocytes.

We observed a wide range of pC/pg RNA levels in our cohort that were elevated in ENCH; in agreement with a report from Suslov and colleagues [10]. We found several polymorphisms in the BCP region, notably in positions 1762 and 1764, that were previously reported to associate with pC/pg RNA [27] and which encode amino acid changes in HBx [25]. These nucleotides have been implicated in the stability of a looped branch in the 3′ end of the RNA, a key regulator of RNA half-life [36]. However, we did not observe any association between these polymorphisms and steady-state pC/pg RNA levels in this cohort.

Peripheral HBsAg has been reported to act as an immune decoy by binding anti-HBs antibodies [37] and associates with functional defects in HBs-specific B and T cells, where continuous stimulation leads to their exhaustion and deletion (reviewed in [38]). As preS1/S2 transcripts are predominantly derived from integrants, this immune dysfunction may persist even in patients with low or inactive cccDNA [13,39]. Therefore, monitoring the loss of peripheral HBsAg, as used in the current definition of functional cure [40], may underestimate the efficacy of new treatments targeting cccDNA. This conclusion is in line with a study from Erken and colleagues, who found that levels of iDNA associate with peripheral HBV DNA, HBsAg, and ALT levels, but not with cccDNA abundance nor its transcriptional activity [41]. We observed an association between iDNA-derived RNAs and age, which may inform future personalised anti-viral approaches.

Viral replication in CHB associates with inflammatory responses and liver damage [42]. We identified 24 inflammatory gene transcripts that associate with cccDNA transcription but found no link with preS1/S2 RNA levels. We recently reported that hypoxia-inducible factors (HIFs), known to be upregulated during inflammation [43], bind and activate HBV cccDNA and yet have minimal impact on the transcription of viral integrants [32], suggesting a potential mechanism for increased cccDNA activity in the inflamed liver. Our observation that none of the inflammation-associated gene transcripts associated with reduced viral replication may reflect HBV adaptation to persist and replicate in a chronic immune environment. The recent discovery that many cancers amplify oncogene transcripts via extra chromosomal circular DNA (ecDNA) [44] suggests a convergent evolutionary pathway for the inflammatory responses that regulate episomal DNA.

We recognise the limitations of bulk PCR approaches to measure viral RNAs that assume similar amplification efficiencies. Our estimates of PCR efficiency show comparable amplification with all primer pairs (Supplementary Figure S4) [20]. Primer mismatches with diverse viral genotypes could lead to a reduced efficiency; however, the primers used in this study were specific for HBV genotype D, shared by all members of our cohort. One approach to address potential bias is digital droplet PCR (ddPCR) and it is reassuring to see consistent observations between our study and that of Prakash et al., who used this method [24]. Several studies have identified chimeric human-viral transcripts where HBV integrates in the proximity of promoters or inter/intra- genic region (reviewed in [45]). Our PCR approach cannot discriminate between chimeric 5’-human-HBV-3’ RNA transcripts and authentic viral RNAs. The limited amount of RNA available from the liver biopsies precluded sequencing approaches to measure transcripts. Despite this caveat, our study shows increased cccDNA transcription during periods of inflammation.

We cannot discriminate whether active HBV replication induces inflammatory responses or vice versa. However, we note that sixteen of the 24 genes in our inflammatory signature (CXCL9, CXCL10, STAT1, STAT2, FXYD2, C7, NLRP3, HLA-DRA, BCL6, C4A, IL10RB, MX1, OAS2, RAF1, RIPK1, and CCL19) were previously identified in the context of HBV infection. The chemokines CXCL9 and CXCL10 have been linked with inflammatory flares, HBV replication, and fibrosis progression (reviewed in [42]). The transcription factor STAT1 is involved in type I interferon signalling and is repressed by HBV [46]. Screening a published scRNA human liver atlas from noninfected subjects allowed us to infer the intrahepatic cell types that express these genes. Although we cannot infer the relative contribution of each cell population in the context of liver disease, we noted that KCs and sinusoidal endothelial cell populations expressed high levels of these genes. Whilst the role of KCs in the persistence of HBV infection is unclear, several studies suggest an anti-inflammatory phenotype that may promote HBV replication (reviewed in [47]). However, culturing KCs with HBV infected hepatocytes showed a reduction in virus replication that was mediated by soluble factors, notably IL-6, IL-10 and tumor necrosis factor [48], illustrating the complexities of recapitulating the chronic liver environment *ex vivo*. Sinusoidal endothelial cells have been reported to tolerise T cells [1] and their activation with NOD-like receptor agonists induced T-cell immunity in vitro and limited HBV DNA and HBsAg in a murine model [49,50]. Our study shows an association between HBV cccDNA transcription and hepatic inflammation that may reflect viral adaptation to persist and replicate in an immune environment that could inform future therapies for hepatitis B and other persistent viral infections.

## Data Availability

All data are available in the main text or the Supplementary Materials.

## Supplementary Materials

The following supporting information can be downloaded at: https://www.mdpi.com/article/10.3390/v14051070/s1, Table S1: Detailed patient information; Figure S1: quantification of HBV RNA copies by different methods or RT approaches; Figure S2: cccDNA copies in biopsies from ENCI and ENCH. Figure S3: BCP sequences. Figure S4: Characterization of RR primers. Figure S5: HBsAg and HbcAg staining patterns in liver biopsies. Figure S6: Cluster analysis of 24 gene set stratifies patients according to ENCI or ENCH grouping. Figure S7: Expression of the 24 inflammatory gene signature.
