# Supplementary material for: Inflammatory Gene Expression Associates with Hepatitis B Virus cccDNA- but Not Integrant-Derived Transcripts in HBeAg Negative Disease
Source: Viruses. 2022 May 17;14(5):1070. doi: 10.3390/v14051070 (PMC9146050; doi:10.3390/v14051070)
Supplement: Supplementary file 1 [file viruses-14-01070-s001.zip › viruses-1660068-supplementary.pdf]

Supplementary Table S1: Detailed patient information.

Code	Sex	Age at Biopsy	EASL	ALT ‡	Staging	Grading	HbsAg §	HBV_DNA #	pgRNA *	Liver HBV_DNA *	cccDNA *†	HBs positivity ¶	HBc Positivity ¶
571	m	62	ENCI	20	0	0		8.50E+02	1.36E+04	4.82E+04	6.40E+01	2	0
576	m	40	ENCH	40	4	4		6.10E+04	5.81E+06	9.28E+04	1.60E+02		
581	m	43	ENCH	40.8	5	3	51.00	5.50E+03	8.77E+02	1.09E+05		1	0
582	f	77	ENCI	20	0	0	1200.00	6.94E+03	6.40E+03	4.10E+04		2	0
584	m	49	ENCI	32	1	1	0.10	4.90E+01	1.49E+02	4.22E+04		0	0
586	m	63	ENCH	90	3	5		1.10E+08	3.35E+07	5.84E+06	1.08E+03		
590	m	60	ENCH	136	6	7	1800.00	9.00E+00	7.11E+06	4.80E+06	1.57E+03	1	3
591	m	86	ENCI	32	1	2	0.13	2.80E+02	1.43E+02	1.44E+04	2.42E+01	0	0
1603	m	50	ENCH	123	5	7		5.30E+06	8.35E+08	1.35E+05	4.00E+02		
1604	f	58	ENCH	80	5	5	2600.00	4.77E+05	6.15E+04	6.06E+04	3.69E+01	1	2
1608	f	62	ENCH	60	6	3	69.00	2.00E+02	3.11E+04	1.66E+04		0	0
1613	m	35	ENCH	40	4	4	4700.00	8.00E+00	2.84E+04	3.75E+04		2	0
1615	f	67	ENCH	160	2	2	1000.00	6.50E+03	1.17E+04	3.94E+04		2	0
1618	f	63	ENCH	60	3	2	860.00	1.60E+05	1.27E+05	3.28E+04	5.95E+01	3	1
1627	f	52	ENCI	44	1	0	220.00	3.70E+02	2.04E+04	1.42E+03	1.60E+02	2	0
1629	m	50	ENCH	40	3	2	19000.00	1.50E+01	1.04E+03	3.90E+04		1	0
1643	m	59	ENCI	33	1	1	1900.00	3.40E+02	1.38E+05	1.48E+04			
1654	m	53	ENCI	40	2	1		5.22E+03	4.97E+03	3.26E+03	2.62E+01	0	0
1661	m	52	ENCH	40	2	2		1.35E+06	8.09E+03	7.42E+04		1	0
1663	m	44	ENCH	160	5	3	8400.00	4.00E+03	1.08E+05	1.68E+05	2.32E+02	2	2
1669	m	38	ENCH	48	1	1	2100.00	8.30E+03	3.63E+04	2.35E+04		2	0
1673	m	54	ENCI	40	1	1	604.00	1.60E+03	4.11E+03	1.80E+04		2	0
1677	f	36	ENCH	32	2	0	3500.00	1.50E+04	4.00E+01	3.04E+04	1.69E+01	0	0
1685	m	70	ENCI	22	2	1		5.20E+02	1.28E+04	9.37E+03		1	0
1710	m	50	ENCI	32	1	3	840.00	1.89E+03	4.38E+03	1.38E+04		1	0
1724	f	52	ENCH	71	3	6	230.00	2.02E+02	8.40E+04	7.92E+03	1.64E+01	1	0

§: HBsAg is measured as IU/ml. Only available for 19 samples.
*: Expressed as normalised copies/μg
†: Only detectable in 13 samples
‡: ALT are expressed as IU/L
#: HBV DNA is measured as IU/mL
¶: HBs/c scores are calculated as % of positive cells: 0= neg, 1<33%, 33%<2<66%, 3>66%

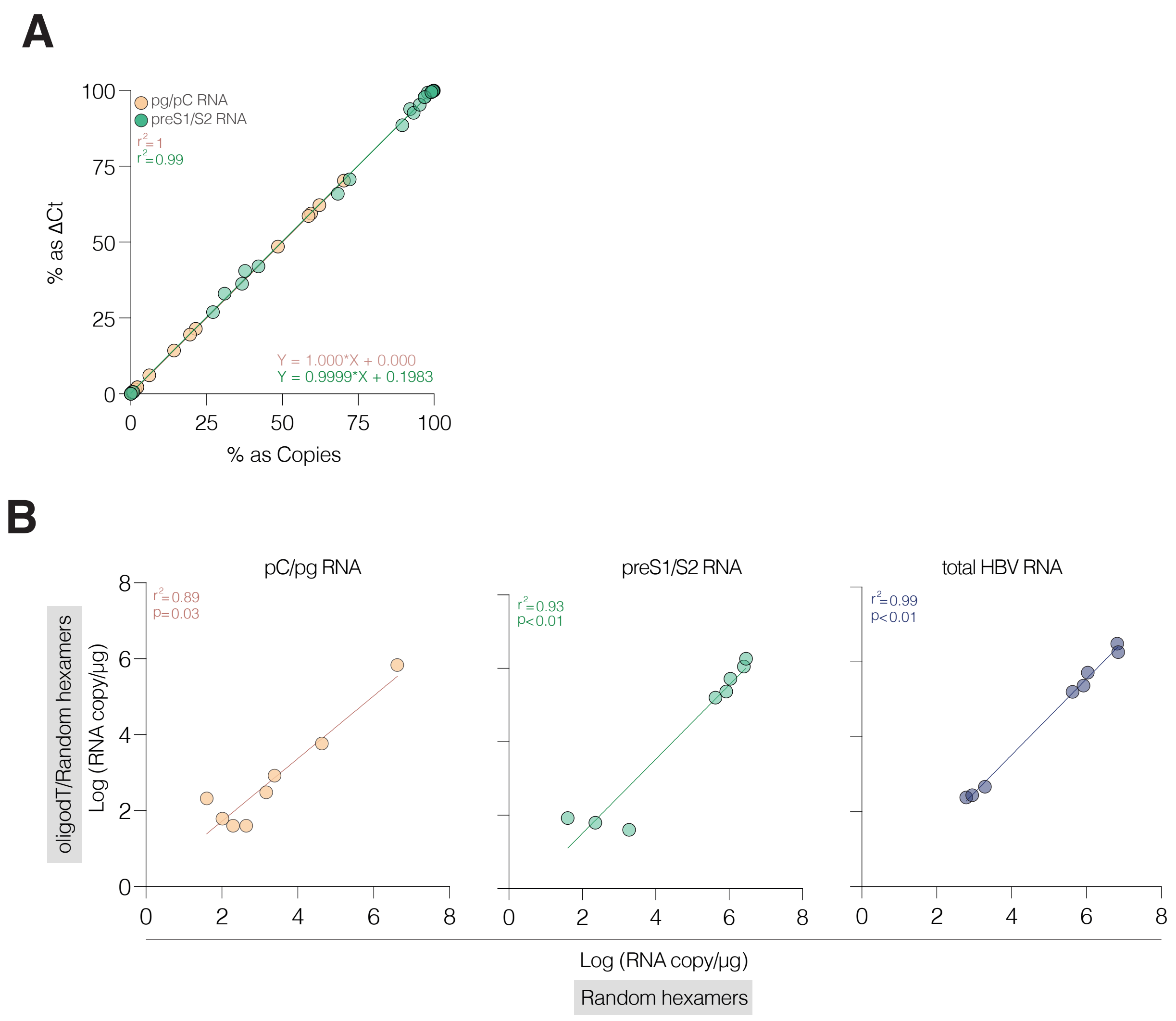

Supplementary Figure S1: (A) Correlation between Delta Ct and Copy number estimation for HBV pC/pg and preS1/S2 RNAs. pC/pg and preS1/S2 RNA copies in the biopsy samples were inferred using the Delta Ct method or copy number and relative abundance compared by Spearman correlation. (B) Correlations between copy number estimations for HBV pC/pg, preS1/S2 and total RNAs obtained with oligo-dT and/or random hexamers- based RT approach. Total HBV RNA primers were designed to target a conserved region in the 3' terminus of all transcripts (1521-1596 relative to the EcoRI site).

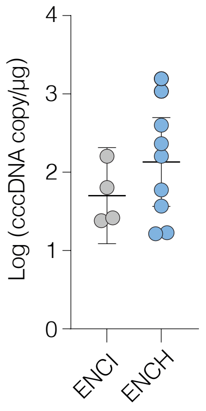

Supplementary Figure S2. cccDNA copies in biopsies from ENCI and ENCH.

	1751	1752	1753	1754	1755	1756	1757	1758	1759	1760	1761	1762	1763	1764	1765	1766	1767	1768	1769
571	G	A	T	T	A	G	G	T	T	A	A	A	G	G	T	C	T	T	T
576	G	M	T	T	A	G	A	T	T	A	A	A	G	R	T	C	T	T	T
581	G	A	T	T	A	G	G	T	T	A	A	A	G	G	T	C	T	T	T
582	G	A	T	T	A	G	A	T	T	A	A	A	G	G	T	C	T	T	T
584	G	A	T	T	A	G	G	T	T	A	A	A	G	G	T	C	T	T	T
586	G	C	T	T	A	G	A	T	T	A	A	A	G	A	T	C	T	T	T
590	G	A	C	T	A	G	A	T	T	A	A	T	G	A	T	C	T	T	T
591	G	A	T	T	A	G	G	T	T	A	A	A	G	G	T	C	T	T	T
1603	G	C	T	T	A	G	A	T	T	A	A	A	G	A	T	C	T	T	T
1604	-	-	-	-	-	-	-	-	-	-	-	-	-	-	-	C	T	T	T
1608	-	-	-	-	-	-	-	-	-	-	-	-	-	-	-	-	-	-	-
1613	G	A	T	T	A	G	A	T	T	A	A	A	G	T	T	G	T	T	T
1615	G	A	C	T	A	G	G	T	T	A	A	A	G	G	T	C	T	T	T
1618	G	A	C	T	A	G	A	T	T	A	C	T	G	A	T	C	T	T	T
1627	G	M	T	T	A	G	A	T	T	A	A	A	G	A	T	C	T	T	T
1629	G	A	T	T	A	G	G	T	T	A	A	A	G	G	T	C	T	T	T
1643	G	A	T	T	A	G	G	T	T	A	A	A	G	G	T	C	T	T	T
1654	G	A	T	K	A	G	G	T	T	A	A	A	G	G	T	C	T	T	T
1661	G	C	T	T	A	G	A	T	T	A	A	A	G	A	T	C	T	T	T
1663	G	A	Y	T	A	G	A	T	T	A	A	A	G	R	T	C	T	T	T
1669	G	A	T	T	A	G	A	T	T	A	A	A	G	G	T	C	T	T	T
1673	G	C	T	T	A	G	A	T	T	A	A	W	G	R	T	C	T	T	T
1677	G	A	C	T	A	G	G	T	T	A	A	T	G	A	T	T	T	T	T
1685	G	A	T	T	A	G	G	T	T	A	A	A	G	G	T	C	T	T	T
1710	G	A	T	T	A	G	G	T	T	A	A	A	G	G	T	C	T	T	T
1724	G	A	T	T	A	G	G	T	T	A	A	A	G	G	T	C	T	T	T

Supplementary Figure S3: BCP sequences. The BCP region (600bp) was PCR amplified from RNA isolated from all patient biopsies and Sanger sequenced in both directions. Consensus sequences were generated with a conservation threshold of 90%.

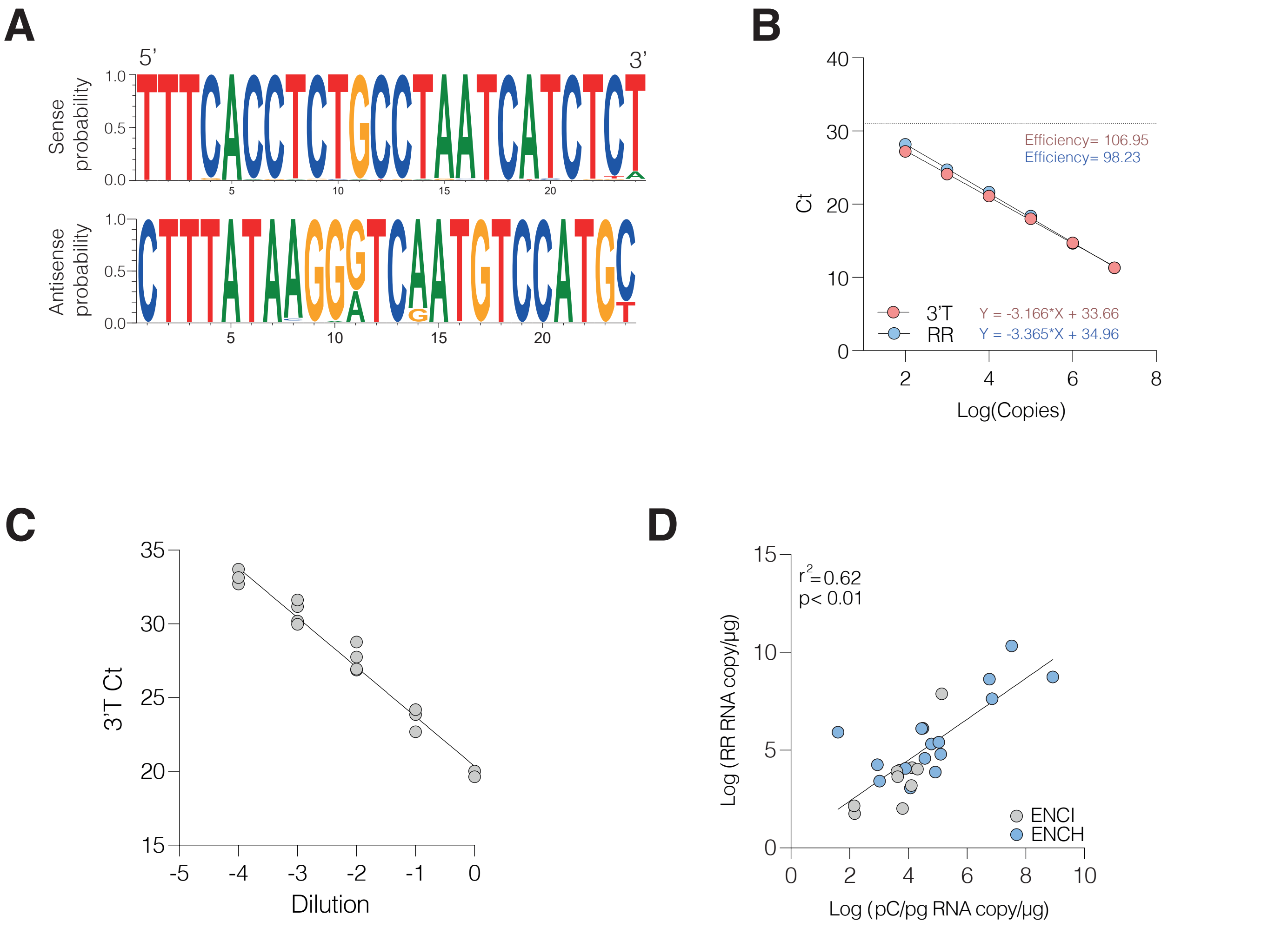

	Slope	Efficiency (%)	Target
T1	-3.06	112.23	pC/pg
T3	-3.175	106.52	pC/pg+preS1/S2
3'T	-3.358	98.52	HBV RNAs
RR	-3.153	107.57	cccDNA-derived RNAs

Supplementary Figure S4: Characterization of RR primers. Sense and anti-sense conservation on 1202 genotype D deposited sequences (hbvdb.lyon.inserm.fr/HBVdb) (A). RR and 3'T amplification efficiency on a HBV1.0 plasmid with 10-fold serial dilutions of known copy numbers (B). cDNA from 4 different biopsies was amplified using 10-fold serial dilutions to calculate slope and qPCR efficiency for each primer pairs and a representative plot with 3'T primer is shown. (C). Correlation between pg/pC RNA copies and cccDNA-derived transcripts in patients according to ENCI (grey) or ENCH (blue) grouping (D).

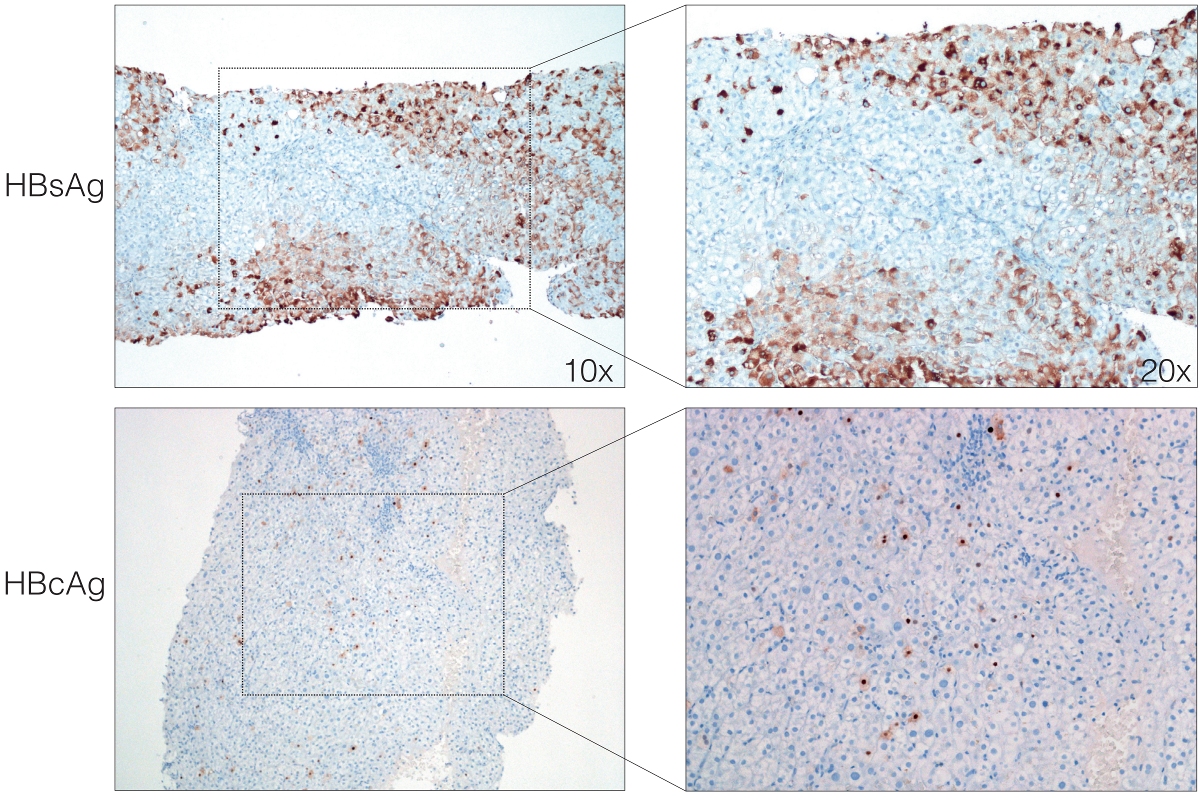

Supplementary Figure S5: HBsAg and HBcAg staining patterns in liver biopsies. HBsAg and HBcAg staining of sequential sections of a representative patient shown at 10 and 20x magnification.

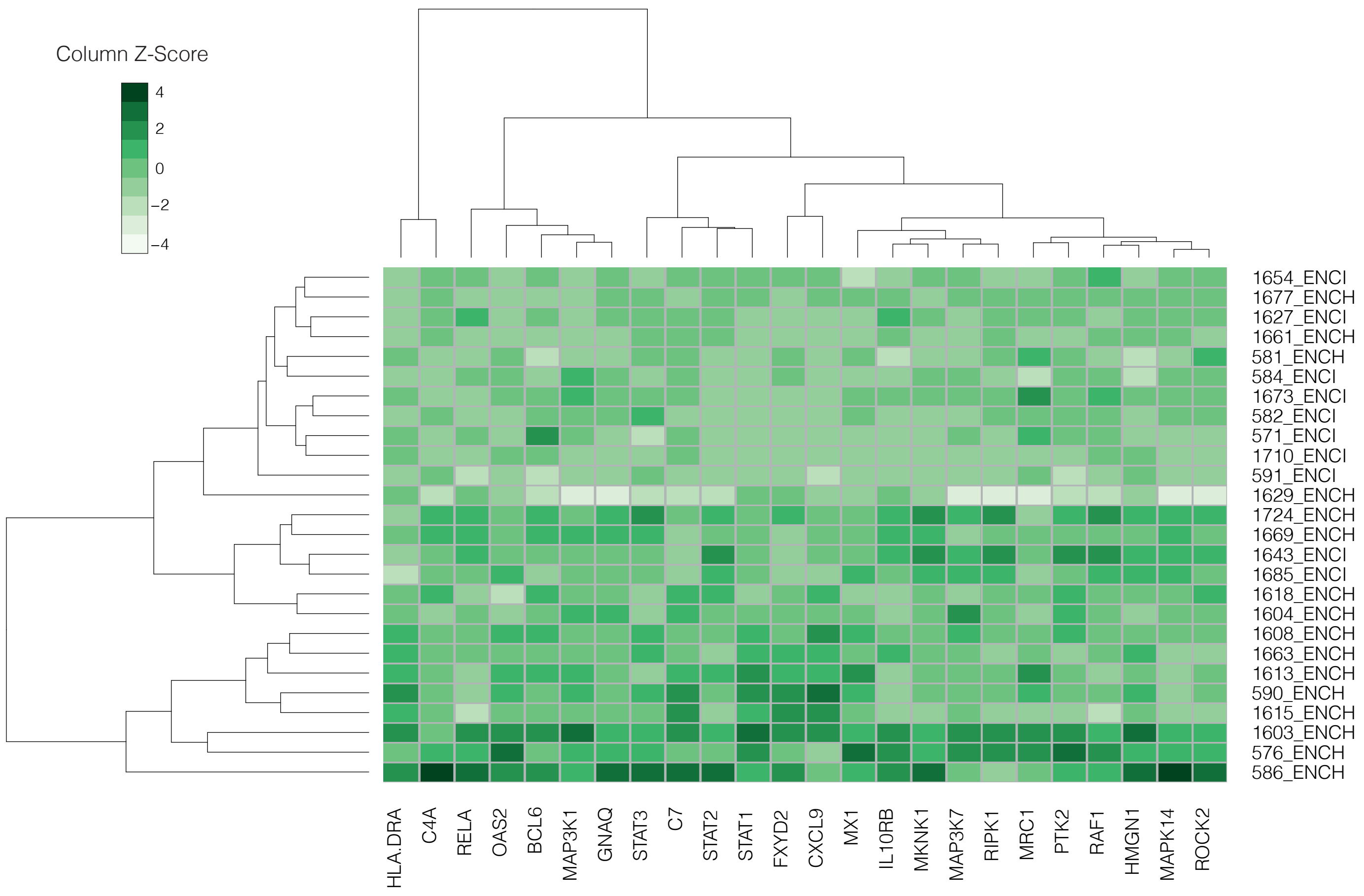

Supplementary Figure S6: Cluster analysis of 24 gene set stratifies patients according to ENCI or ENCH grouping.

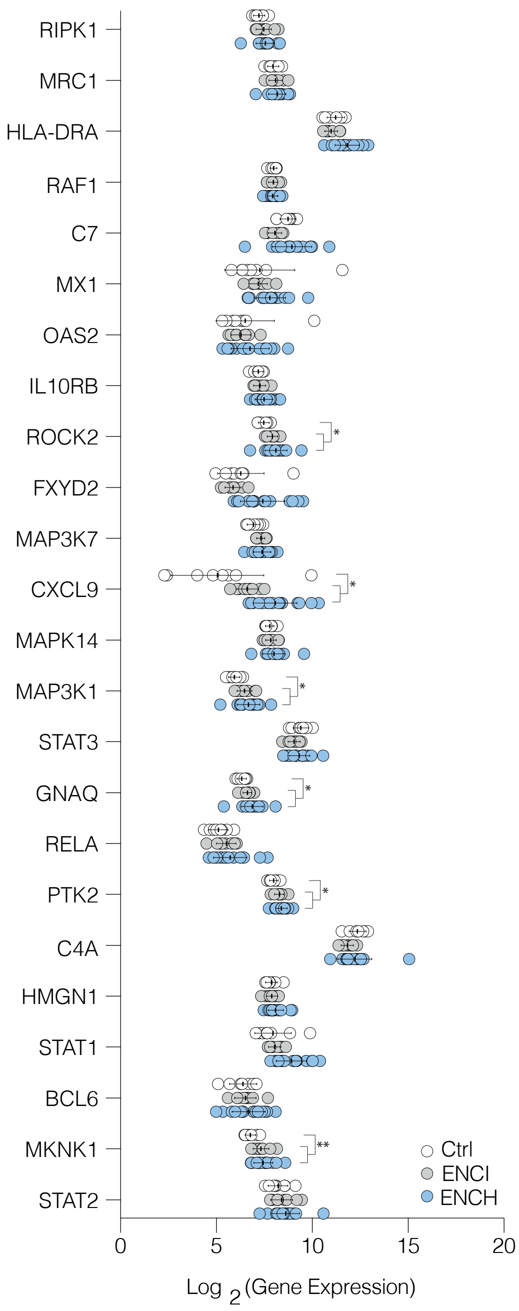

Supplementary Figure S7: Expression of the 24 inflammatory gene signature. Log2-transformed normalized counts shown for non-viral controls, ENCI and ENCH. Mann-Whitney U test between Ctrl and CHB, with Holm-Sidak pvalue correction.
